# TUBE Project: Transport-Derived Ultrafines and the Brain Effects

**DOI:** 10.3390/ijerph19010311

**Published:** 2021-12-28

**Authors:** Maria-Viola Martikainen, Päivi Aakko-Saksa, Lenie van den Broek, Flemming R. Cassee, Roxana O. Carare, Sweelin Chew, Andras Dinnyes, Rosalba Giugno, Katja M. Kanninen, Tarja Malm, Ala Muala, Maiken Nedergaard, Anna Oudin, Pedro Oyola, Tobias V. Pfeiffer, Topi Rönkkö, Sanna Saarikoski, Thomas Sandström, Roel P. F. Schins, Jan Topinka, Mo Yang, Xiaowen Zeng, Remco H. S. Westerink, Pasi I. Jalava

**Affiliations:** 1 Department of Environmental and Biological Sciences, University of Eastern Finland, 70210 Kuopio, Finland; moyang@uef.fi (M.Y.); pasi.jalava@uef.fi (P.I.J.); 2 VTT Technical Research Centre of Finland Ltd., 02044 Espoo, Finland; paivi.aakko-saksa@vtt.fi; 3 Mimetas BV, 2312 BZ Oegstgeest, The Netherlands; l.vandenbroek@mimetas.com; 4 Centre for Sustainability, Environment and Health, National Institute for Public Health and the Environment (RIVM), 3721 MA Bilthoven, The Netherlands; flemming.cassee@rivm.nl; 5 Institute for Risk Assessment Sciences (IRAS), Utrecht University, 3508 TD Utrecht, The Netherlands; r.westerink@uu.nl; 6 Faculty of Medicine, University of Southampton, Southampton SO17 1BJ, UK; R.O.Carare@soton.ac.uk; 7 A.I. Virtanen Institute for Molecular Sciences, University of Eastern Finland, 70211 Kuopio, Finland; sweelin.chew@gmail.com (S.C.); katja.kanninen@uef.fi (K.M.K.); tarja.malm@uef.fi (T.M.); 8 Biotalentum Ltd., 2100 Godollo, Hungary; andras.dinnyes@biotalentum.hu; 9 Computer Science Department, University of Verona, 37129 Verona, Italy; rosalba.giugno@univr.it; 10 Department of Public Health and Clinical Medicine, Division of Medicine/Respiratory Medicine, Umeå University, 901 87 Umea, Sweden; ala.muala@umu.se (A.M.); anna.oudin@umu.se (A.O.); thomas.sandstrom@umu.se (T.S.); 11 Center for Translational Neuromedicine, Faculty of Health and Medical Sciences, University of Copenhagen, 2200 Copenhagen, Denmark; nedergaard@sund.ku.dk; 12 Centro Mario Molina Chile, Strategic Studies Department, Santiago 602, Chile; poyola@cmmolina.cl; 13 VSParticle B.V., 2629 JD Delft, The Netherlands; t.pfeiffer@vsparticle.com; 14 Aerosol Physics Laboratory, Physics Unit, Faculty of Engineering and Natural Sciences, Tampere University, 33720 Tampere, Finland; topi.ronkko@tuni.fi; 15 Atmospheric Composition Research, Finnish Meteorological Institute, 00101 Helsinki, Finland; sanna.saarikoski@fmi.fi; 16 IUF—Leibniz Research Institute for Environmental Medicine, 40225 Dusseldorf, Germany; Roel.Schins@IUF-Duesseldorf.de; 17 Department of Genetic Toxicology and Epigenetics, Institute of Experimental Medicine of the CAS, Videnska 1083, 142 20 Prague, Czech Republic; jan.topinka@iem.cas.cz; 18 Guangdong Provincial Engineering Technology Research Center of Environmental Pollution and Health Risk Assessment, Department of Occupational and Environmental Health, School of Public Health, Sun Yat-sen University, Guangzhou 510080, China; zxw63@mail.sysu.edu.cn

**Keywords:** air pollution, brain, particulate matter, UFP, toxicology, CNS, traffic

## Abstract

The adverse effects of air pollutants on the respiratory and cardiovascular systems are unquestionable. However, in recent years, indications of effects beyond these organ systems have become more evident. Traffic-related air pollution has been linked with neurological diseases, exacerbated cognitive dysfunction, and Alzheimer’s disease. However, the exact air pollutant compositions and exposure scenarios leading to these adverse health effects are not known. Although several components of air pollution may be at play, recent experimental studies point to a key role of ultrafine particles (UFPs). While the importance of UFPs has been recognized, almost nothing is known about the smallest fraction of UFPs, and only >23 nm emissions are regulated in the EU. Moreover, the role of the semivolatile fraction of the emissions has been neglected. The Transport-Derived Ultrafines and the Brain Effects (TUBE) project will increase knowledge on harmful ultrafine air pollutants, as well as semivolatile compounds related to adverse health effects. By including all the major current combustion and emission control technologies, the TUBE project aims to provide new information on the adverse health effects of current traffic, as well as information for decision makers to develop more effective emission legislation. Most importantly, the TUBE project will include adverse health effects beyond the respiratory system; TUBE will assess how air pollution affects the brain and how air pollution particles might be removed from the brain. The purpose of this report is to describe the TUBE project, its background, and its goals.

## 1. Introduction

The WHO estimates that 99% of the global population breathes air containing high levels of pollutants, especially particulate matter (PM), exceeding its guideline limits [1]. It has already been established that the respiratory system is the main target organ for air pollution and that particles deposit throughout the entire respiratory tract depending on their aerodynamic size and morphology [2,3]. Recent investigations provide evidence that particles can be translocated from the lungs into the circulation and secondary target organs [4,5]. According to current knowledge, the translocation of particles occurs mainly through endocytosis by alveolar epithelial cells [6]. Very little is known about the translocation of particles from other parts of the respiratory system, e.g., the olfactory epithelium. While the adverse effects of air pollutants on the lungs and the cardiovascular system are undisputed [1,7], in recent years, indications of effects beyond the lungs and circulatory system have become more evident.

Neurological diseases and exacerbated cognitive dysfunction have already been shown to be associated with air pollution [8,9,10,11,12] and, in particular, living in the vicinity of roads with high traffic density [13,14]. Furthermore, air pollution is linked with Alzheimer’s disease (AD) [14,15], the most common neurodegenerative disease and the main cause of dementia in the elderly. Some mechanisms have been revealed. Chronic exposure to complex mixtures of air pollutants in urban environments has been shown to induce toxicological effects in the brain, including chronic inflammation, oxidative stress, loss of protein homeostasis, and disruption of the blood–brain barrier (BBB) [16,17,18,19,20]. Clearance is critical to limit the adverse effects induced by pollutants; however, it is not known how and if solid particles are cleared from the brain. While the association of air pollutants with cognitive decline and neurodegenerative diseases has been discussed, it remains unclear which components of air pollution are responsible for these adverse effects and what are the molecular and cellular mechanisms underlying the connection between brain health, AD, and air pollution.

Although several components of air pollution may be at play, recent experimental studies point to a key role of ultrafine particles (UFPs), the smallest size fraction of particles that can be inhaled [21]. Anthropogenic UFPs are predominantly combustion derived and characterized by a mobility diameter < 100 nm [22]. They are usually byproducts of processes involving industrial and traffic-related combustion. Both the type of fuel and the mode of combustion determine the characteristics of UFPs, including chemical composition, particle size, surface area, and solubility. In Europe, road transport contributes to total UFPs of around 32% in Greece to about 97% in Luxemburg [23]. Emissions from road traffic can enter car, bus, and truck cabins [24], affect pedestrians, cyclists, etc., and thus cause high levels of personal exposure [25]. The concentration of road-traffic-derived UFPs has continued to rise to date, and despite the increasing number of electric vehicles and better filtration technologies, nonexhaust emissions remain substantial. It is well established that inhalation exposure to UFPs causes lung and systemic inflammation and oxidative stress [21]. UFPs have several characteristics, such as distribution by diffusion and a very small size, by which they may cause diverse adverse effects on the respiratory system and circulation, independent of the larger particle sizes. Due to this, UFPs may be able to change cellular function by circumventing normal signaling pathways. Moreover, UFPs have the ability to enter cells and cause DNA damage [26]. Recent advances in transportation engine technologies, despite reducing particulate mass emissions, could also lead to some unforeseen adverse health effects. For example, particle number concentrations (PNCs) in modern technologies are often rather high, although the mass and size of the emitted particles are very small. In addition to particles, traffic also causes emission of gaseous volatile organic compounds (VOCs) and semivolatile organic compounds (SVOCs). It is also unknown which sources, such as gasoline and diesel exhaust, brake and tire wear, road dust, and compositions of traffic emissions, cause the adverse effects seen in epidemiological studies related to the central nervous system.

While only UFPs that are solid, nonvolatile particles with a size > 23 nm are included in the current emission legislative limit in the EU, the toxic effect may also be associated with the semivolatile fraction. Moreover, very little is known about the effects of extremely fine (<10 nm) particles and of (S)VOCs from combustion engines. Their harmful effects on humans, especially on the health of the brain, are relatively little studied, even though UFPs can cross bodily barriers and can enter the brain. There is an urgent need to understand the interplay of air pollutants with adverse effects in the brain in order to guide political decision making for the efficient reduction of air pollutants. This could, in the long run, reduce the economic burden caused by diseases associated with them. 

**Figure 1:**
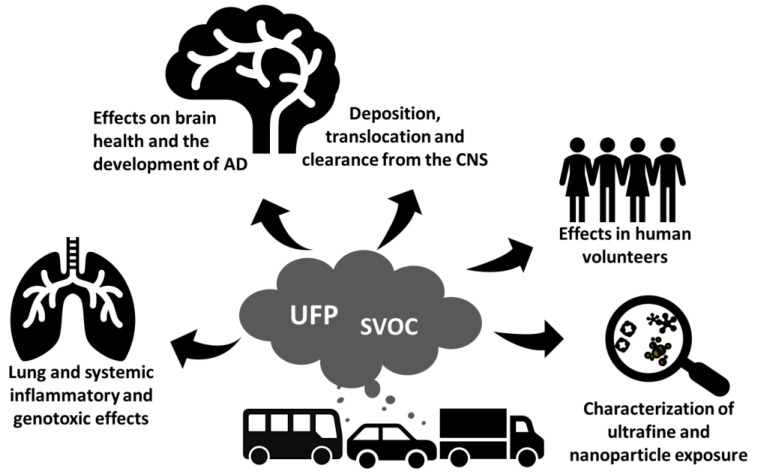
Schematic figure of the TUBE project. Abbreviations: AD: Alzheimer’s Disease, CNS: central nervous system, UFP; ultrafine particles, SVOC: semivolatile organic compounds.

To address this unmet need, the Transport-Derived Ultrafines and the Brain Effects (TUBE) project funded by the EU-Horizon 2020 brings together interdisciplinary expertise that aims to unravel fundamental questions of traffic-related air pollutants and the health of the brain (Figure 1). The main objective of the TUBE project is to generate critical information for the risk assessment of traffic-related UFPs and their implication in the pathogenesis of AD. TUBE research seeks to reveal new biomarkers of UFP exposure effects on brain health and the development of AD and leverages on new in vitro human-cell-based models and in mice.

Employing state-of-the-art research methodologies, TUBE intends to:

1. Reveal the harmful components of air pollution, especially UFPs and emissions from engines operated with fuels of high aromatic content.
2. Cover representative exhaust emissions from road traffic, including heavy-duty diesel and diesel and gasoline cars, representing the latest emission control technologies.
3. Gather data on unregulated emissions, aromatics, and polyaromatic hydrocarbons, as well as nitrogen-containing compounds in gaseous SVOCs or the particulate phase of exhausts.
4. Collect UFP concentration and size distribution measurements from across the globe (Europe, Chile, China) to assess the effects of differences in emission regulations, societal structure, and air quality policies.
5. Identify which air pollutants are responsible for the adverse health effects seen in humans by using in vitro and in vivo models to study the effects of UFPs on the respiratory system and the brain.
6. Identify biomarkers for the early detection of brain diseases related to air pollution.
7. Provide mitigation strategies for emissions of road traffic and nonroad equipment and provide data that will be used to support planning the future of the traffic policies.
8. Collaborate with other EU-Horizon 2020 projects toward the same goal of reducing ultrafine air pollutants.

In this report, we will describe the TUBE project, its background, and its goals in more detail.

## 2. Ultrafine and Nanoparticle Exposure Characterization with Sampling for Biological Research of Different Transport Mode Sources and Fuel Aromatic Matrix

In the TUBE project, advanced instrumentation and measurement methods are used for the characterization of exhaust aerosols, including, e.g., determination of the chemical composition of emitted particles, the lung deposited surface area (LDSA) of particles, and the size distribution of particles down to the nanometer scale, exploited in laboratory experiments for emissions from different engines and vehicles. In these experiments, special focus is on the emission effects of fuel properties and, on the other hand, the size and volatility of emitted aerosols. 

While existing scientific information underlines the role of ambient air pollution in human population health, information related to the spatial differences in UFP concentrations and the characteristics of UFPs in different environments is scarce. For example, although recent studies have shown that traffic can emit significant amounts of extremely small particles (<30 nm) into the urban atmosphere [27], routine monitoring is currently not required by legislation in any of the EU member states. Moreover, although particle emission regulations limit the emission of many traffic modes with respect to solid particles larger than 23 nm, information regarding their atmospheric concentrations is incomplete or suboptimal. This causes challenges both in the evaluation of the effects of technological trends on ambient UFP concentrations and in the preparation of future political actions aiming to improve air quality, human health, and environmental safety.

In the TUBE project, the approach is to build investigations using in vitro and in vivo models on the understanding of UFPs in real environments. This will aid the development of the research chain from engine exhaust to cell, animal, and human exposure studies so that the interpretation of health-related results can be made with respect to different traffic modes and real urban environments. This approach includes the characterization of UFPs in various different urban environments using a mobile aerosol laboratory (ATMo-Lab [28]) equipped with instrumentation suitable for measurements of ambient particle size distributions and concentrations down a to few nanometers, as well as for the determination of particle composition and solid particle fraction. Mobile measurements are complemented by stationary measurements for ambient UFPs in traffic-influenced environments and linked with research on the technologies to be used in nanoparticle sampling.

The first results of studies on ambient aerosols were published by Salo et al. [27]. Based on their experimental observations, particle size distribution and particle concentrations can differ significantly between the roadside environments of relatively clean and highly polluted cities, i.e., Helsinki and the Delhi area. Regarding size distributions, the most significant differences were seen in the size distributions of the LDSA of ambient particles. In the city with a lower ambient air particle mass (Helsinki), the corresponding LDSA had smaller particle sizes. In Helsinki, the median particle size of the LDSA size distribution was 80 nm when the local air quality was not significantly affected by long-range-transported particles, while in the roadside environment in the Delhi area, it was as high as 410 nm. This can have implications on how particulate matter affects human health in these environments since, in addition to particle concentrations, particle composition itself depends on particle size. In the TUBE project, we build on existing knowledge for the research of other urban environments, especially in Europe, South America, and China. 

Toxic emission species are potentially carried by particles and condensed SVOCs, as discussed by Aakko-Saksa et al. [29]. Some species, for example polyaromatic compounds, can induce carcinogenic and mutagenic effects, while others, such as reactive oxygen, nitrogen compounds, and metals, can lead to inflammation and tissue damage. In laboratory experiments with cars and diesel engines using fuels of different aromatic contents, advanced characterization of exhaust samples is combined with toxicological studies. In addition to direct exposure to exhausts with an air–liquid interface (ALI), exhaust particle and SVOC samples are collected for toxicological studies conducted by partners of the TUBE project.

## 3. Lung and Systemic Inflammatory Effects and Genotoxicity Caused by Nanoparticles Related to Different Traffic Modes

In the TUBE project, we are developing several in vitro models that will increase the relevance of cell models compared to animal models and improve our understanding of the mechanisms of the toxicity of UFPs and exhaust emissions. With the information gathered from the project, it will be possible to use these models to replace some of the experiments performed in animals in future inhalation toxicology research.

The lungs are the major exposure and target organ for air pollution and the first barrier to potential effects in secondary organs and tissues, including the brain. Exposure of the lungs to UFPs from different traffic modes in the environment may lead to acute and chronic lung or systemic inflammation. Although the exact mechanisms by which UFPs induce lung and systemic proinflammatory effects are still not completely understood, the importance of oxidative stress is now well recognized [7,30,31]. The ability of UFPs to trigger oxidative stress has been linked to their ROS-generating properties, resulting from their specific physiochemical properties that are highly dependent on their source. For instance, in urban environments, UFPs are often characterized by a carbon core covered with transition metals and organic hydrocarbons. As such, ROS can be formed from their high specific surface area, Fenton-like reactions, and quinone redox cycling processes [7,32]. ROS may be generated from cellular sources within and beyond the lungs, including the oxidative burst from activated macrophages and neutrophils and NOX enzymes in epithelial and endothelial cells [33]. As a consequence, UFPs can modulate the concentrations of intracellular calcium and activate transcription factors, leading to increased proinflammatory cytokine production [34,35]. UFPs can aggravate lung inflammation related to bacterial endotoxins, although the highest concentration of these proinflammatory lipopolysaccharides is predominantly found in coarse particles [36], measured also from smaller size fractions [37]. The ROS-generating and inflammatory properties of inhaled UFPs are also considered to drive genotoxic effects in the lungs via the formation of oxidative DNA lesions [38,39]. In addition, UFPs can carry mutagenic compounds deep into the lungs, which, depending on their bioavailability, can cause genotoxicity by the formation of bulky DNA lesions [40,41]. 

The importance of the fraction of UFPs in the adverse cardiopulmonary effects of ambient air pollution particles is increasingly recognized and supported by epidemiological observations [42]. However, more research is needed to better understand the qualitative and quantitative impacts of the fraction of UFPs on lung and systemic inflammation and genotoxicity. Ongoing and future developments in vehicle technologies and transportation policies are anticipated to influence the likelihood of exposure to UFPs in decreasing size distribution. The effects of UFPs have not been sufficiently covered in previous studies due to shortcomings in currently available state-of-the-art technologies. For example, small UFPs are difficult to collect from traffic environments for toxicological experiments, and in ALI exposure methods, the deposition efficiency of UFPs has been rather poor.

The TUBE project aims to disentangle these shortcomings by developing methods for inhalation toxicology experiments using novel approaches for both cells and animals. For example, a 3D microfluidic lung-on-a-chip model for inhalation toxicity testing will be developed. Moreover, both in vitro and in vivo inhalation toxicology experiments on traffic-related emissions in the source environment and in the laboratory will be conducted to estimate the toxicological effects of different components of air pollutant mixtures on the lungs and beyond. The aim of these investigations is to unravel the genotoxic and tissue remodeling effects in the lungs, as well as the systemic inflammatory effects of traffic-related air pollutants.

A major shortcoming of the previous literature is that a large share of the experiments was conducted using outdated engine and emission control technologies. Within the TUBE project, we compare different modern technologies and fuels in lung cell models by using both collected particles and ALI exposures. In ALI exposures, cells, e.g., A549, THP-1, and Calu-3, are directly exposed to diluted emissions, and comparison of different technologies is possible in conditions more reminiscent of real life. In ALI exposures, aerosols do not change from their original form, unlike in submerged exposures conducted with filter-collected particles [43,44]. In addition, studying gaseous compounds is possible by using ALI methods [45]. However, ALI methods cannot capture all the features present in in vivo experiments, and the expressions of responses can be different [46,47]. For example, they lack feedback mechanisms, the solubility and biological activity of the exposures are not the same as in living organisms. ALI methods can overcome the major weaknesses of submerged cell culture methods, e.g., selective exposure to insoluble and soluble components, coagulation of particles, and loss of semivolatile or volatile components and gases. They have proven to be highly usable for studying very different forms and sources of aerosols (e.g., [48,49,50]). The designs of different ALI systems are highly different and usually meant to meet specific study questions. However, in vitro ALI methods are not yet possible for each cell model. Therefore, particulate samples are collected for several purposes at the same time in order to perform offline experiments, i.e., by testing the collected samples after controlled resuspension in various in vitro assays. In addition to particle fraction, semivolatile compounds are collected at the same time. Interestingly, those compounds have proved to cause rather strong effects in cell models compared to particles (unpublished data).

In addition to the evaluation of the aforementioned “real life” UFPs and engine emissions, the TUBE project will perform experiments on very small model particles, each with a defined chemical composition, obtained by innovative particle generator devices. This complementary approach allows for the investigation of the toxicological relevance of the defined size subfractions of UFPs, specifically regarding their ability to trigger inflammatory and genotoxic responses. 

To overcome oversimplified cell models, new organ-on-a-chip models, based on air-lifted human primary lung epithelium and microfluidics, have been developed. Within the project, only selected inhalation experiments will be performed in mice, with the specific aim to investigate the inflammatory and genotoxic properties and underlying mechanisms of the very smallest fraction of UFPs.

## 4. Effects of Traffic-Derived UFPs on Brain Health and the Development of Alzheimer’s Disease

The TUBE project aims to provide critical information for the risk assessment of UFPs in the context of AD. Specifically, this includes the characterization of the effects of UFPs on neuroinflammatory responses in human-induced pluripotent stem-cell-derived microglia, assessment of AD-related phenotypes in patient-derived olfactory mucosal cells, and identification of UFP exposure on neurotoxicity and development of AD in in vitro brain organoids [51] and mouse models (5XFAD, APP/PS1). Overall, this research will generate critical new knowledge on both the in vitro and in vivo health effects of UFPs and increase our understanding of the biological processes leading to their effects.

The brain is one of the most vulnerable organs in the human body, potentially targeted by soluble and insoluble molecules, with deleterious consequences. Accumulating evidence indicates that exposure to air pollutants has the potential to modify cerebral function, and exposure to air pollution is connected to AD [52]. Epidemiological studies have already linked living in highly polluted areas to AD [14]; however, the impact of UFPs on AD-related phenotypes has not been assessed in detail on the cellular and molecular level. 

Although some effects of air pollution on cells of the olfactory system (main route of entry of UFPs), blood–brain barrier (BBB), and microglia have been studied and summarized [12,19], the effects of UFPs on brain health and function are largely unknown. For example, the BBB limits the entry of harmful substances into the brain parenchyma; however, small and often highly lipophilic molecules of a particular composition can pass through the BBB [53]. Another protective feature limiting the effects of harmful substances on the brain is the microglia, which, as resident immune cells of the brain, are able to respond to the alterations that they encounter. Microglia can modulate neuronal firing, and alterations to microglial homeostatic activities can result in neuronal malfunctions [54]. Accumulation of toxic molecules, such as beta-amyloid in AD, induce microglial overactivation, thereby promoting disease development [55]. Larger air pollutant particles have already been shown to induce microglial activation [19]; however, little is known about how UFPs affect these processes. In order to promote brain health, and make brain disease prevention and treatment a clinical reality, transdisciplinary research is urgently needed to truly understand how air pollution shapes brain health and how exactly it is associated with AD. To discover the cellular impact of traffic-related UFPs and how they are implicated in AD pathogenesis, epigenetic, transcriptomic, and functional data will be generated from human neurons, microglia [56], and olfactory mucosal cells [57].

## 5. Ultrafine Particle Deposition to, Translocation in and Clearance from the Central Nervous System

The TUBE project aims to unravel UFP deposition, translocation, and clearance in the brain; demonstrate whether dural lymphatic vessels participate in the clearance of UFPs from the brain; and analyze how UFPs influence the clearance of amyloid-beta through the intramural periarterial drainage pathway. Furthermore, TUBE aims to demonstrate how UFPs influence glymphatic clearance and investigate if particles can be cleared through the glymphatic system along the interstitial waste product pathways. 

**Figure 2:**
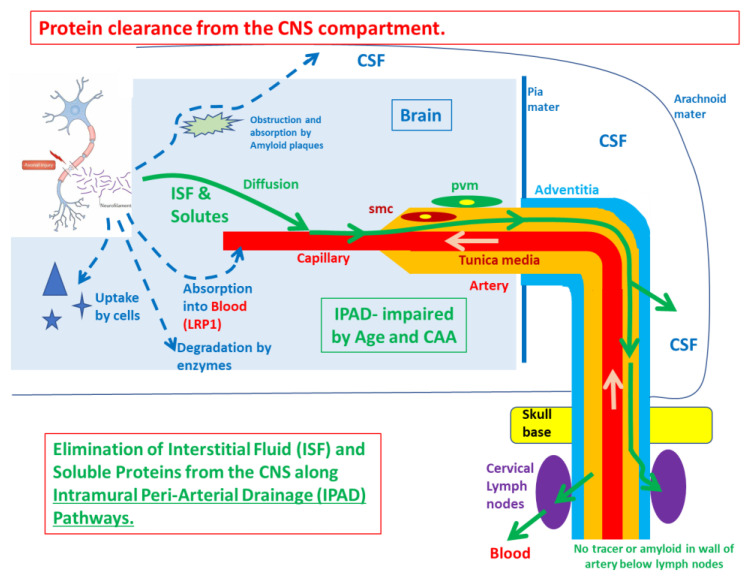
Mechanisms of protein clearance from the CNS compartment. Abbreviations: CAA: cerebral amyloid angiopathy, CSF: cerebrospinal fluid, ISF: interstitial fluid, LRP: Low density lipoprotein receptor-related protein, IPAD: intramural periarterial drainage.

Proper clearance of redundant and undesired molecules and particles from the brain is vital for brain functions. Apart from blood, there are two fluids in the brain: interstitial fluid (ISF) and cerebrospinal fluid (CSF). They have different origins and mechanisms of drainage (Figure 2). ISF not only fills the narrow gaps in the central nervous system (CNS) and functions to deliver nutrients to brain cells but also facilitates the clearance of waste from neural metabolism. ISF drains along the basement membranes of capillaries and arteries as intramural periarterial drainage (IPAD) [58]. CSF enters the brain parenchyma as convective influx/glymphatic drainage. CSF fills the ventricular system of the brain and is produced by the choroid plexus where it flows through the ventricles, reaching into the subarachnoid spaces [59,60]. From the subarachnoid space, CSF drains into the peripheral lymphatic system. Failure of clearance of ISF leads to the deposition of amyloid in the walls of blood vessels as cerebral amyloid angiopathy (CAA) and AD. Deficits in the proper clearance of the CNS metabolic waste and toxic molecules render the brain vulnerable to neurodegeneration, including the development of AD. Failure of IPAD with increasing age is associated with the failure of elimination of fluid and toxic amyloid-beta (Aβ) from the brain, leading to CAA and AD [61]. 

Inhalation of air pollutants may lead to UFPs reaching the brain parenchyma. Evidence from epidemiological and experimental studies has shown that inhaled polluted air accelerates dementia and amyloid plaque formation [13,62]. It is possible that UFPs may reach the subarachnoid channels surrounding the olfactory nerve, effectively the same channels that drain CSF into the nasal mucosa and cervical lymph nodes. Once they reach the CSF, solutes and particles may access the cerebral parenchyma and thereby cause deleterious effects on brain cell functions. Depending on the size of the UFPs, they may either remain in the parenchyma or drain through the routes that are typically used for the elimination of proteins from the brain. There is no information in the literature on if and how UFP particles are cleared from the brain parenchyma. Thus, it is completely unknown whether the particles leave the brain within the waste disposal systems of the CNS or rather localize and accumulate into the brain parenchyma. In addition, UFPs may interfere or even block the clearance pathways of the CNS, thus rendering the brain vulnerable to the accumulation of waste and toxic molecules, including AD pathological proteins. On the other hand, if UFPs drain via the brain fluid drainage pathways, the adverse effects of UFPs on brain health may be therapeutically targeted by enhancing the fluid clearance pathways.

## 6. Effects of Traffic-Related Ultrafine Particles in Human Volunteers

The TUBE project aims to clarify the impact of traffic-related nanoparticles in humans both by using experimental (human volunteers) and epidemiological study approaches. The effects of both short-term and long-term exposure to air pollution will be assessed. TUBE will use the data gathered by the Betula study from northern Sweden, which has been described in detail elsewhere [63], to evaluate the impact of PMs of a specific size on the risk of developing AD. TUBE also aims to evaluate how UFPs influence brain functions in healthy individuals by conducting chamber studies. This will yield important information on the short-term effects of UFPs on cognitive and olfactory functions. Moreover, the TUBE project has the possibility to include biomarkers from a cohort study in the Chinese megacity Guangzhou. The TUBE project will also evaluate the levels of metal oxides or other relevant biomarkers in the blood of AD patients to reveal their possible association with AD. 

The link between long-term exposure to air pollution, reduced cognitive function [14], and dementia [64,65,66] has already been established. Based on these and other studies, the Lancet commission added air pollution to their list of modifiable risk factors for AD in 2020 [67]. While epidemiological studies have already established a connection between air pollution and neuronal decline, associations specifically between particles of different sizes and AD need to be further investigated. Furthermore, although studies have generally concentrated on the effects of long-term high exposure on health, it is also important to assess long-term effects in low-exposure areas. In addition, it is unclear if there are any short-term effects of air pollution on cognitive function, even though some studies suggest so [68,69,70].

Air pollution exposure may also be a risk factor for decreased olfactory function [71]. Although a decrease in olfactory function is an important and well-known risk factor for AD, to date, there are only a few studies on the potentially complex associations between exposure to ambient particles, AD, and olfactory function. The olfactory system may be particularly vulnerable to air pollution, as it is directly exposed to air pollutants. 

## 7. Risk Assessment and Policy Recommendations

While UFPs are known to induce a range of adverse health effects, knowledge on how traffic-related UFPs affect lung-, neuro-, olfactory-, and genotoxicity is currently insufficient. Additionally, the exact levels and duration of exposure that lead to adverse effects still need to be determined. Ongoing and future research focused on the in vitro and in vivo health effects of UFPs and the comparison with human volunteer and epidemiological studies, including human biomarker analysis, will allow for mechanism-based risk assessment approaches. These combined data will provide a scientific basis to derive intervention measures to improve air quality and reduce harmful emissions that will aid future traffic policy.

As a first step toward mechanism-based human risk assessment, it is critical to generate concentration–dose–effect relationships for effects of UFPs on toxicity in the lungs, olfactory system, and brain using in vitro and in vivo models and to derive no observed adverse effect levels (NOAELs) or benchmark dose levels (BMDL) for the various toxicity endpoints. Such NOELs and BMDLs can then be used to estimate the internal dose at the target organ(s) by integration of blood levels with exposure levels and information on UFP deposition, translocation, and clearance in the lungs and nervous system and determine the margin of exposure (MOE) or margin of safety (MOS). MOE is equivalent to MOS and is a ratio of the NOAEL (from in vitro or in vivo studies) and the estimated (internal) dose at the human target organ: MOS = NOAEL/Estimated (internal) human exposure dose. Taking into account uncertainty factors, an MOS ≥ 100 is generally considered to be sufficiently protective. Notably, it is possible that in some geographical areas, the MOE or MOS will be smaller than 100 or even smaller than 1, indicating that human exposure levels will exceed the levels at which effects on human health are to be expected, i.e., the exposure risk is not acceptable from a toxicological point of view. In such instances, exposure reduction measures need to be introduced to prevent adverse health effects. Importantly, results from in vitro and in vivo experiments allow for comparing risks in relation to qualitative and/or quantitative differences in exposure (e.g., high vs low aromatic fuels, differences in the contribution of UFPs in exhausts and ambient air, different size ranges of UFPs, specific emission factors). Combined with information from human volunteer and epidemiological studies, this approach will reveal if exposure to traffic-related UFPs at environmentally relevant levels may actually lead to adverse health effects in the lungs, olfactory system, and brain, and if so, which specific factors (components) present the largest risk. Subsequently, exposure reduction measures for these specific factors and if exposure reduction measures should be considered to minimize effects on public and environmental health. By this, giving insight on e.g., why living near to traffic can lead to impaired cognition and dementia can be foreseen.

Notably, current legislative limits include only solid, nonvolatile particles > 23 nm. Moreover, current (EU) air quality legislation does not require measurements of the UFPs. For these reasons, it is highly important to include measurements of UFPs and semivolatile components or BC into health-related studies on air pollution. In fact, all of these are mentioned by WHOs updated air quality guidelines [1]. Previous studies showing effects of larger particle sizes include UFPs within PM2.5 or PM10. However, usually, the methodology for detecting the smallest particle fractions has been insufficient, and the same metrics do not apply for UFPs. Instead, particle surface area and number concentrations are better metrics than PM mass, which is highly dominated by larger particles. At the same time, the emissions from traffic are under a constant change. For example, the new engine and aftertreatment technologies have been very effective in reducing the emission mass concentration, but the number of concentrations of the UFPs has been increasing at the same time [72]. Identification of qualitative and quantitative contributions of traffic-related extremely fine particles, including UFPs < 23 nm, on adverse health effects will thus support mitigation actions and strategies by regulatory bodies and policy makers. Since air quality is also closely linked to the climate of the earth, policies aiming to reduce UFP, therefore, may lower the burden of disease attributable to air pollution and contribute to the mitigation of climate change e.g., by reducing black carbon concentrations, which are known SLCFs [73].

## 8. Conclusions

Air pollution is one of the leading health burdens globally, and the adverse effects of air pollutants beyond their effects on the respiratory and cardiovascular systems, in particular on the nervous system, are still largely unresolved. Furthermore, the effects of the smallest fraction of ultrafine particles and the effects of SVOCs are still unknown, even though in reducing overall air pollution levels, the exposure to these has become proportionally more important. Recent study outcomes indicate that UFPs might have serious adverse health effects that are not well controlled by current air quality standards [21]. This suggests that there is an urgent need to fully investigate the effects and to consider setting emission and exposure limits.

The overarching goal of the TUBE project is to use novel methods for detecting UFP and SVOCs from the traffic environments and engine exhausts and link them with the adverse effects in the respiratory system and particularly in the brain. Importantly, the project will yield new information on unregulated nanoparticles with an aerodynamic size less than 23 nm, even down to 1 nm. TUBE will provide knowledge on which emission sources or source environments provide the greatest exposure to the smallest UFPs, which are anticipated to cause severe effects beyond lung exposure in humans and are particularly concerning regarding genotoxicity. The TUBE project will help to understand the interplay of traffic-related air pollutants with inflammatory, cytotoxic, and genotoxic effects in the lungs, as well as beyond in the brain, in particular in the pathogenesis of neurodegenerative diseases such as AD.

In the longer term, these key findings could be implemented into future regulatory requirements for emission characterization and innovative toxicity testing. These findings will eventually lead to new standards and limit values for the traffic-related emissions in engines and in urban environments. Through the implementation of the TUBE project, European competitiveness in the fields of environmental science and neuroscience is advanced. The project is designed to give suggestions on what emissions are the most important for future regulations to protect human health. Moreover, the large matrix of studied samples, fuels, aftertreatment systems, and endpoints gives an overview of the technological choices that could benefit the health of citizens. By demonstrating the detrimental effects of air pollutants on brain health, TUBE is expected to steer political decision making toward reducing air pollution by providing both the means and the need to measure ultrafine particles and thereby reduce the number of working days lost due to illness and yearly premature deaths.

## Data Availability

Not applicable.
